# Antibacterial activity of the stem bark of *Tieghemella Heckelii* Pierre ex. A Chev against methicillin-resistant *Staphylococcus aureus*

**DOI:** 10.1186/s12906-017-1681-8

**Published:** 2017-03-27

**Authors:** B. G. Kipre, N.K. Guessennd, M.W. Koné, V. Gbonon, J. K. Coulibaly, M. Dosso

**Affiliations:** 1 Institut Pasteur Côte d’Ivoire, Département de Bactériologie/Virologie, Centre National de Reference Antibiotiques, 3 Avenue de l’Université Cocody, B.P 490, Abidjan 01, Côte d’Ivoire; 2 Université Nangui Abrogoua Côte d’Ivoire, B.P 801, Abidjan 01, Côte d’Ivoire; 3 Centre Suisse de Recherches Scientifiques en Côte d’Ivoire, Km 17, Dabou, Côte d’Ivoire

**Keywords:** *Tieghemella heckelii*, Methicillin-resistant *Staphylococcus aureus*, Antibacterial, Côte d’Ivoire

## Abstract

**Background:**

*Tieghemella heckelii* (Sapotaceae) is a medicinal plant used in Africa, particularly in Côte d’Ivoire for treating various diseases including infections. Identification of prospective antibacterial compounds from stem bark of this plant as a result of its medicinal virtue, led to screening activity against methicillin resistant bacteria.

**Methods:**

Six extracts (hexane, chloroform, ethyl acetate, ethanol, methanol and sterile distilled water) were prepared and tested on methicillin resistant *Staphylococcus aureus* (MRSA) using broth microdilution method for activity assessment. From this experiment, the minimum inhibitory concentrations (MICs) and minimum bactericidal concentrations (MBCs) of the plant extracts were determined in sterile 96-well microplates in order to search for both bacteriostatic and bactericidal effects. Afterwards, data analysis was performed using GraphPad Prism5 software (One-way ANOVA and Turkey Multiple Comparison test). The results were then presented as Mean ± SD for experiment repeated three times.

**Results:**

Four extracts (ethyl acetate, methanol, ethanol and sterile distilled water) showed credible potency, with strong, significant, and moderate growth inhibition of the MRSA tested. The MIC values which varied from 45 μg/mL to 97 μg/mL according to microbial phenotype, resolutely established the activity of the plant extracts. Additionally, the MBC values which varied, depending on the type of bacteria strain, revealed the bacteriostatic and bactericidal effects of the active extracts against Methicillin-resistant *Staphylococcus aureus*.

**Conclusion:**

The present study is a confirmation of the therapeutic potential of *Tieghemella heckelii* and its promising contribution to the discovery of a novel antibacterial drug pertaining to these resistant strains.

## Background

Among the gram positive bacteria, *Staphylococcus aureus* plays a crucial role in community and nosocomial infections. To fight against these bacteria, the beta-lactams therapeutic class is mostly used in human medicine [1]. The molecular structure of these compounds could be described by a beta-lactam ring attached to variable side chains, and cycles. In fact, the pharmacokinetics, along with the antibacterial activity of each antibiotic of this group depend upon the structure of the attached side chains and cycle. Unfortunately, development of several bacteria-resistance mechanisms has caused inactivation of available current antibiotics. Consequently, community settings are endangered, for the pharmaceutical industry holds as at now, few new antibiotics to offer.

The emergence or re-emergence of bacteria-resistance has therefore become a global health concern, and public health menace in Sub-Saharan Africa, especially in Côte d’Ivoire, where methicillin resistance prevalence rate was around 34.5% in 2014 [2]. On the contrary, a great number of resistant bacteria strains were identified as seriously challenging current antimicrobial drugs [3]. In order to address this issue, natural products are solicited to play an incredible role, as an alternative to eradicate the multi-drug resistance [4]. For, in Africa more than 80% of the populations use plants as medication towards infections, and 25% of the prescriptions against infectious diseases worldwide are plant-based. In this context, early studies have investigated some plants used in folk medicine [5]. Nevertheless, records in the literature do not show data on antibacterial activity of *Tieghemella heckelii* (Sapotaceae) [6].

The present study, therefore aims at screening the *in vitro* assessment of this activity towards MRSA, as well as promoting the Ivorian national flora.

## Methods

### Plant material

The plant material used was the stem bark of *Tieghemella heckelii* Pierre ex. A Chev*.* (Sapotaceae). The plant species was authenticated at the herbarium of “Centre Suisse de Recherches Scientifiques en Côte d’Ivoire, Adiopodoumé (Abidjan-Côte d’Ivoire)”, and registered under Voucher number 3021. The stem bark was collected within the period of September to October 2014 in the Haut Sassandra, a mid-west region in Côte d’Ivoire.

### Bacteria strains

Bacteria tested and their antibiogram, were provided respectively by the strains collection bank (Bio-bank) and the National Center for Antibiotics Reference at Institut Pasteur Côte d’Ivoire. These Extended-Spectrum Beta-Lactamases (ESBLs) harboring isolates were made up of six methicillin-resistant *Staphylococcus aureus* (MRSA). Additionally, American Type Collection Culture (ATCC) strains were used as reference material.

### Methods

#### Preparation of extracts

The stem bark was shade dried over 15 days, and powdered in a mortar. Then, the extracts were prepared by macerating successively 200 g of plant powder in 1 L of the different solvents of increasing polarity. Extraction was carried out for 48 h for each solvent used. After filtration successively on hydrophilic cotton and filter paper, the extracts were dried in an oven at 40 °C to yield a dense residue. Each extract sample was then transferred to a glass vial until use.

## Antimicrobial assays

The different plant extracts were serially diluted from 0.048 mg/mL to 12.5 mg/mL separately in both sterile 96-well microplates, and test tubes using broth microdilution method [7]. Suspension (50 μL) was added to each well, and potency (MICs) was evaluated [8]; that is the lowest concentration of plant extract that completely inhibited the growth of the bacterium in the well or test tube [9]. Then, the MBC values were determined by sub-culturing the samples with no visible growth in the MIC assays. For this purpose, the inoculum was diluted from 10^−1^ to 10^−4^ in test tubes, and streak-seeded with a calibrated loop (2 μL) on Muller-Hinton agar. The set-up included bacterial growth controls containing the test inoculum (50 μL) and the negative controls without bacterial inoculum. Extracts controls were likewise included into the set-up. The first batch of Petri dishes containing the agar were labelled A, and incubated at 37 °C for 24 h. Then, after reading the MIC values, the tube content which did not show bacteria growth, was streak-seeded on Muller-Hinton agar. This second batch was labelled B, and the MBCs were determined by comparing bacteria growth in A and B. The extract is said to be bacteriostatic if the ratio MBC: MIC is equal to 4, whereas it is said to be bactericidal when it is equal to 2.

## Results

The Ethanol extract, which showed strong growth inhibition for bacteria 408C/14, 1541C/14, 485C/14 and 446C/14, exhibited a moderate trend for bacteria 1000C/14, and weak activity towards 499C/14. The potency of the extract was demonstrated at a minimum inhibition concentration value of 0.390 mg/mL, by displaying a bacteriostatic effect for 446C/14, 408C/14, and a bactericidal activity against 1541C/14 and 485C/14. Activity was also observed for bacteria 499C/14 and 1000C/14 respectively at MIC 1.562 and 0.781 mg/mL (Table 1). Additionally, the Turkey Multiple Comparison showed significant variation (*P* < 0.05) of the crude extract efficacy within the range of 0.048 mg/mL to 12.5 mg/mL. Another feature that confirmed this trend is the one-way analysis of variance which showed a pronounced means difference of the efficacy with an R squared value of 0.6326.

**Table 1:** Antibacterial activity of crude ethanol extract of *Tieghemella heckelii* on Methicillin-resistant and reference strains of *Staphylococcus aureus*

Strains	MIC	MBC	MBC/MIC	Effect
408C/14	0,390 ± 0.001	1562 ± 0.001	4	Bacteriostatic
1000C/14	0,781 ± 0.001	1562 ± 0.001	2	Bactericidal
1541C/14	0,390 ± 0.001	0,781 ± 0.001	2	Bactericidal
485C/14	0,390 ± 0.001	0,781 ± 0.001	2	Bactericidal
499C/14	1562 ± 0.001	3125 ± 0.001	2	Bactericidal
446C/14	0,390 ± 0.001	1562 ± 0.001	4	Bacteriostatic
ATCCC 25923	0,781 ± 0.001	1562 ± 0.001	2	Bactericidal

*MIC* minimum inhibitory concentration, *MBC* minimum bactericidal concentration, values are expressed as mean ± SD of 3 experiments (ANOVA and Turkey test)

Evaluation of the ethyl acetate extract showed growth inhibition of all MRSA tested with a MIC figure of 0.097 mg/mL for strains 408/C, 499C/14, 1000C/14 and 1541C/14. The plant extract has also demonstrated antibacterial activity towards strains 446C/14, and 485C/14 with MIC respective values of 0.195 mg/mL and 0.781 mg/mL (Table 2). Using the Turkey Multiple Comparison, the MIC means difference was significant for both strains 446C/14 and 485C/14. This was confirmed by the one-way analysis of variance with an R squared value of 0.6519.

**Table 2:** Antibacterial activity of ethyl acetate extract of *Tieghemella heckelii* on Methicillin- resistant and reference strains of *Staphylococcus aureus*

Strain	MIC	MBC	MBC/MIC	Effect
408C/14	0,097 ± 0.003	0,097 ± 0.003	1	Bactericidal
1000C/14	0,097 ± 0.003	0,390 ± 0.001	4	Bacteriostatic
1541C/14	0,097 ± 0.003	0,195 ± 0.005	2	Bactericidal
485C/14	0,781 ± 0.001	1.562 ± 0.001	2	Bactericidal
499C/14	0,390 ± 0.001	0,390 ± 0.001	1	Bactericidal
446C/14	0,195 ± 0.005	0,390 ± 0.001	2	Bactericidal
ATCC 25923	0,097 ± 0.003	0,195 ± 0.005	2	Bactericidal

*MIC* minimum inhibitory concentration, *MBC* minimum bactericidal concentration, values are expressed as mean ± SD of 3 experiments (ANOVA and Turkey test)

Methanol extract mixed with different MRSA inoculum strongly prevented the bacteria growth with MIC highest value of 0.048 mg/mL for bacterium 1000C/14 and 0.097 mg/ml for strains 408C/14, 485C/14, and 499C/14. For 1541C/14, the extract displayed moderate anti-MRSA potency at MIC value of 0.781 mg/mL with a bactericidal effect, and weak activity at 1.562 mg/mL for 446C/14 with a bacteriostatic effect (Table 3). The statistical analysis also showed significant variation of MIC means difference within the extract concentrations range of 0.048 to 12.5 mg/mL (Turkey Multiple Comparison test), which was confirmed by the one-way analysis of variance (*P* = 0.0003; R^2^ = 0.5987).

**Table 3:** Antibacterial activity of methanol extract of *Tieghemella heckelii* on Methicillin- resistant and reference strains of *Staphylococcus aureus*

Strain	MIC	MBC	MBC/MIC	Effect
408C/14	0,097 ± 0.003	0,195 ± 0.005	2	Bactericidal
1000C/14	0,045 ± 0.001	0,195 ± 0.005	4	Bacteriostatic
1541C/14	0,781 ± 0.001	1562 ± 0.001	2	Bactericidal
485C/14	0,097 ± 0.003	0,195 ± 0.005	2	Bactericidal
499C/14	0,097 ± 0.003	0,097 ± 0.003	1	Bactericidal
446C/14	1562 ± 0.001	6,25 ± 0.001	4	Bacteriostatic
ATCC 25923	0,045 ± 0.001	0,097 ± 0.003	2	Bactericidal

*MIC* minimum inhibitory concentration, *MBC*: minimum bactericidal concentration, values are expressed as mean ± SD of 3experiments (ANOVA and Turkey test)

Residual Aqueous extract tested on MRSA in the screening experiment, revealed growth inhibition for strain 408C/14 with MIC value of 6.25 mg/mL, activity against 1000C/14 with MIC value of 3.125 mg/mL, and growth inhibition for bacteria strains 446C/14, 485C/14, 499C/14 and 1541C/14 with MIC value of 1.562 mg/mL (Table 4). Additionally, the extract displayed a bactericidal effect against all MRSA tested. Ultimately, the Turkey Multiple Comparison test showed significant variation of the extract efficacy within the concentrations range. This was confirmed by the one-way analysis of variance (*P* = 0.1323; R^2^ = 0.2013).

**Table 4:** Antibacterial activity of aqueous extract of *Tieghemella heckelii* on Methicillin- resistant and reference strains of *Staphylococcus aureus*

Strains	MIC	MBC	MBC/MIC	Effect
408C/14	6,25 ± 0.001	12,5 ± 0.001	2	Bactericidal
1000C/14	3125 ± 0.005	6,25 ± 0.001	2	Bactericidal
1541C/14	1562 ± 0.001	3125 ± 0.005	2	Bactericidal
485C/14	1562 ± 0.001	3125 ± 0.005	2	Bactericidal
499C/14	1562 ± 0.001	3125 ± 0.005	2	Bactericidal
446C/14	1562 ± 0.001	3125 ± 0.005	2	Bactericidal
ATCC 25923	3125 ± 0.005	6,25 ± 0.001	2	Bactericidal

*MIC* minimum inhibitory concentration, *MBC* minimum bactericidal concentration, values are expressed as mean ± SD of 3 experiments (ANOVA and Turkey test)

## Discussion

The surveillance of multidrug resistance bacteria is a crucial challenge to the global health scientists. Therefore, the need to promote new natural compounds discovery is to be met for the preservation of humanity. From this perspective, traditional medicine appears to be the way out and as a result, it is practiced worldwide by use of herbal plants for therapeutic purposes [10]. To illustrate, in the West African region, especially in Côte d’Ivoire, people use plants as medications in rural settings [11, 12]. Thus, *Tieghemella heckelii* is locally used for its anti-infectious properties [11]. However, the efficacy of the plant against multiresistant bacteria has not been investigated. Additionally, relatively less expensive antibacterial need to be developed in order to address patients subjected to infectious diseases, regardless of their living standards with the vision to completely eradicate the antibacterial drugs resistance. Early studies reported traditional medicinal plants to exhibit successful activity against methicillin-resistant *Staphylococcus aureus*, a gram-positive bacterium with an outer cell wall permeable to lipophilic solutes [13]. The results of the present study showed that the stem bark of *Tieghemella heckelii* had a great potential value against MRSA. Also, the MIC that ranged from 0.045 mg/mL to 12.5 μg/mL withstood this propriety of the plant part. However, the cutoff point was fixed at a MIC value of 0.0 97 mg/mL. As a result, all other extract exhibiting figures above the MIC aforementioned against the bacteria strains was not considered. To illustrate, from the prospective four active extracts, both the ethyl acetate and methanolic extracts displayed highly strong antibacterial activity because they exhibited MIC lowest values of 97 μg/mL and 45 μg/mL. But, methanol extract appears to contain the most promising antibacterial agent (MIC 45 μg/mL). Additionally, plant extracts showed inhibition concentrations lower than that reported elsewhere. In comparison with earlier antibacterial activity investigation, the extracts of the stem bark of *Tieghemella heckelii* showed stronger efficacy against MRSA than the grap-seed extract which exhibited MIC values of 1000 μg/mL to 5000 μg/mL [14]. When compared with studies carried out with *Piper betle* to assess the antibacterial activity, the present evaluation of plant extracts’ efficacy against MRSA showed lower MIC values compared to 78 to 156 μg/mL exhibited by the leaf extracts of *Piper betle* against gram positive *Staphylococcus aureus* [13].

## Conclusion

The screening experiment conducted with the MIC and MBC determination, showed that the stem bark of *Tieghemella heckelii* contains ingredients that prevent the growth of the tested micro-organisms (6 Methicillin-resistant *Staphylococcus aureus*). Therefore, they displayed high antimicrobial properties towards the gram-positive bacteria tested. Additionally, the lower MIC values of 0.048 and 0.097 mg/mL (Methanol and Ethyl Acetate extracts) were respectively recorded on 16.7% and 83.3% of the investigated MRSA species whereas the lower MBC values of 0.097 and 0.195 mg/mL (Ethyl acetate and Methanol extracts) exerted a credible activity on 33.3% and 50.0% of the MRSA. These encouraging results from the Methanol and Ethyl Acetate extracts, brought about the perspective of further molecular purification. Moreover, the lower MBC values of the plant extract raised the toxicity concern and further study was carried out on human cell lines to address this issue.

Consequently, the present study, which revealed the antibacterial properties of the plant extracts, is a justification of the use of *Tieghemella heckelii*’s stem bark to treat some of the tropical infectious diseases in Africa and particularly in Côte d’Ivoire. This fact is withstood by the bactericidal effect of the Residual Aqueous Extract mostly used in traditional medicine. Nevertheless, there is still room for an in depth investigation, in order to make the plant best useful to rural settings and to select it as an alternative to bacteria resistance.

## Abbreviation

ATCC: American Type Collection Culture
DMSO: Dimethyl Sulfoxide
ESBL: Extended-Spectrum Beta-Lactamase
GSE: Grasp-seed extract
MBC: Minimum bactericidal concentration
MIC: Minimum inhibitory concentration
MRSA: Methicillin-resistant *Staphylococcus aureus*.
SD: Standard deviation
